# Fluoride Release by Glass Ionomer Cements, Compomer and Giomer

**Published:** 2009

**Authors:** Sayed Mostafa Mousavinasab, Ian Meyers

**Affiliations:** * Associate Professor, Department of Restorative Dentistry and Torabinejad Dental Research Centre, School of Dentistry, Isfahan University of Medical Sciences, Isalmic Khorasgan Azad University, Isfahan, Iran; ** Post Colgate Chair of General Practice Dentistry. The University of Queensland, Brisbane, Australia

**Keywords:** Compomers, Fluorides, Giomer, Glass ionomer

## Abstract

**Background:**

To measure the amounts of fluoride released from fluoride-containing materials, four glass ionomer cements (Fuji IX, Fuji VII, Fuji IX Extra and Fuji II LC), a compomer (Dyract Extra) and a giomer (Beautifil) were used in this study.

**Methods:**

Twenty cylindrical specimens were prepared from each material. The amount of released fluoride was measured during the first week and on the days 14 and 21 by using specific fluoride electrode and an ionanalyzer. The results were statistically analyzed using analysis of variance (two-way ANOVA) and Tukey Kramer multiple comparison tests (p=0.05).

**Results:**

Significant differences were seen in fluoride release of different days and materials (p<0.05). The maximum cumulative fluoride release of days 1-7 was related to Fuji VII, followed by Fuji IX Extra, Fuji II LC, Fuji IX, Dyract Extra and Beautifil in descending order and this order remained the same until the 21^st^ day.

**Conclusion:**

Fuji IX, Fuji VII, Fuji IX Extra, and Fuji II LC released higher amounts of fluoride compared to Beautifil and Dyract Extra in this study. It seems that the extent of the glass ionomer matrix plays an important role in determining the fluoride releasing ability of glass ionomer cement materials.

## Introduction

There are several fluoride containing dental restorative materials available in the market including glass ionomer cements, resin modified glass ionomers, polyacid modified resins (compomers), giomers and resin composites. Fluoride containing dental materials show clear differences in the fluoride release and uptake characteristics^1^,^2^ and may act as fluoride reservoir to increase fluoride level in saliva, plaque and hard dental tissues, or may help to prevent or reduce secondary caries.^3^–^8^ Short and long term fluoride release from restorative materials are related to their matrices, setting mechanism and fluoride content, nature of fluoride incorporated into resin based materials and also depends on several environmental conditions.^7^,^9^–^11^ The pattern of fluoride release from glass ionomer cements is characterized by an initial rapid release, followed by a rapid reduction in the rate of release of fluoride after short time.^12^–^14^ In an ex-vivo study comparing fluoride release behavior of a conventional glass ionomer (limerick glass) with a resin modified one (Fuji Ortho^TM^ LC), both materials exhibited the classic fluoride release curve of GICs with a more sustained release for conventional one over time.^15^ The ability of glass ionomer sealants to serve as fluoride reservoir in oral cavity and retaining a low fluoride level in oral fluids have been proved in a study.^16^ A recent development has been the introduction of the giomers materials. Variable extent of the GI phase is determined by differences in the resin composition of the restoratives.^17^ Another study has shown that the amount of total and free fluoride release from giomer was higher than that of compomer and composite and concluded that the extent of glass ionomer matrix of the glass filler plays an important role in fluoride releasing and recharging abilities of the resin based materials.^18^ Also it has been shown that giomers and compomers do not have the initial fluoride “burst” effect associated with the glass ionomer cements.^19^ The aim of this study was to examine the fluoride releasing ability of glass ionomer and resin based materials containing fluoridated glass fillers.

## Materials and Methods

The materials tested in this study included four glass ionomer cements, Fuji IX, Fuji VII, Fuji IX Extra, Fuji II LC, a compomer (Dyract Extra) and a giomer (Beautifil). The characteristics of the used materials in the study are given in Table 1.

**Table 1:** Materials used in the study.

Product	Type	Manufacturer	Shade	Code
GC Fuji VII	GC	GC Corporation, Tokyo, Japan	Pink	FVII
GC Fuji IX GP Fast	GC	GC Corporation, Tokyo, Japan	A3	FIX
GC Fuji IX GP Extra	GC	GC Corporation, Tokyo, Japan	A3	FIX EX
GC Fuji II LC	GC	GC Corporation, Tokyo, Japan	A3	FII LC
Dyract Extra	RMGC	Densply Detrey GmbH, Germany	A3	DE
Beautifil	Giomer	Shofo Dental Corporation, USA	A3	BT

GC = Glass Ionomer

### Specimen preparation

Cylindrical aluminum molds (4 mm diameter and 8 mm depth) used to prepare the required samples. The materials prepared according to the manufacturer’s instruction and packed into the molds. The specimen’s top surface was covered by a Mylar strip and glass slides and allowed to set at room temperature for ten minutes in chemically curing materials. The light curing materials cured from top and bottom using a light source (Pencure, J Morita MFG corp., Japan) for 40 s. An additional 20 s light was given in the middle of sample from both sides. Prior to testing, the specimens incubated in a 95% relative humidity environment at 37°C for 24 hours. Then, the specimens of each group (n = 20) immersed in 1 ml deionized water in polyethylene vials and stored in the incubator at 37°C.

### Fluoride release

After 24 hours, the containers were thoroughly shaken, and then the samples removed, dried and returned into a new vial containing 1 ml of deionized water. The procedure repeated daily and then, cumulative fluoride release measurement was made during the first week and at the end of second and third weeks. A fluoride ion selective electrode (Ion Check 45, Radiometer analytical, France) used to measure fluoride release. The instrument calibrated according to manufacturer’s instruction using six standard fluoride solutions containing 0.20, 1.00, 2.00, 10.00, 20.00 and 100 ppm F, respectively. Before measurement, 0.1 ml of TISAB III was added to each solution to provide constant background ionic strength, decomplex fluoride and adjust PH, and then concentration (in ppm) of each sample solution was recorded. The final results reported as fluoride release rate (µg/cm^2^/day) and cumulative fluoride release (µg/cm^2^) taking into account the surface area and solution volume of each specimen using the following equation, mgF/cm2 = ppm (µgF/mL) mL (storage media volume at unit time) 1/ 1.25cm^2^ where 1.25 cm^2^ is the surface area of each tested sample material. The data were analyzed using two-way ANOVA and Tukey Kramer multiple comparison and Student t tests (P = 0.05).

## Results

### Cumulative fluoride release

**Figure 1:**
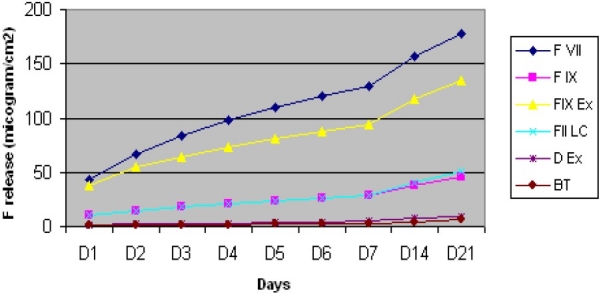
Cumulative fluoride release of the materials.

Analyzing the data showed significant differences in cumulative fluoride release between different days and different materials (P < 0.05). The maximum cumulative fluoride release for days 1-7 was related to Fuji VII, followed by Fuji IX Extra, Fuji II LC, Fuji IX, Dyract Extra (DEX) and Beautifil (BT) in descending order and this order remained the same until the 21^st^ day (Table 2). Fluoride released from FII LC compared to that of FIX on the 1^st^ day was higher, but this difference was not significant. On the day 14 and 21, this trend changed and FIILC released more fluoride compared to FIX, and their fluoride release difference became significant (P < 0.05). All the materials continued to release fluoride, but a higher increase in fluoride release was seen for FVII, FIX, FIX EX and FII LC compared to BT and DEX, after the 7^th^ day. There was a curve divergence between fluoride release of FVII and FIX until the 7^th^ day, but after that the curves were almost parallel (Figure 1). DEX and BT both released low amounts of fluoride, but DEX released more significant fluoride compared to BT on the 1^st^ day (P< 0.05) and this difference was seen in all days of fluoride release (Table 3).

**Table 2:** Cumulative fluoride release from tested materials (µg/cm^2^); standard deviations are given in parenthesis.

Days	D1	D2	D3	D4	D5	D6	D7	D14	D21
**FVII**	42.64 (5.87)	66.52 (8.08)	83.16 (8.48)	97.91 (10.24)	110.06 (11.38)	120.44 (12.15)	128.77 (11.7)	156.85 (13.62)	178.18 (15.15)
**FIX**	10.00 (1.60)	14.96 (2.11)	18.86 (2.46)	21.11 (2.47)	23.95 (2.46)	26.29 (2.61)	28.34 (2.69)	37.71 (3.02)	45.99 (3.74)
**F IX EX**	38.39 (9.11)	55.18 (10.29)	64.50 (10.96)	73.56 (11.66)	80.91 (12.02)	88.17 (12.81)	94.23 (13.33)	117.52 (15.4)	134.37 (16.4)
**F II LC**	10.52 (1.69)	14.59 (1.68)	18.52 (1.83)	21.41 (1.98)	23.98 (2.10)	26.31 (2.11)	28.99 (2.16)	40.75 (2.63)	51.63 (2.70)
**DEX**	1.42 (0.41)	2.07 (0.53)	2.74 (0.58)	3.20 (0.58)	3.60 (0.55)	4.20 (0.59)	4.60 (0.56)	7.55 (0.69)	9.62 (0.75)
**BT**	0.76 (0.28)	1.24 (0.39)	1.55 (0.39)	1.84 (0.39)	2.16 (0.42)	2.42 (0.43)	2.77 (0.46)	4.43 (0.57)	5.95 (0.72)

**Table 3:** Mean fluoride release of the tested materials in relation to different days. SD is given within parenthesis.

Days	D1	D7	D14	D21
Materials				
**FVII**	42.64 (5.87)	128.77 (11.7)	156.85 (13.62)	178.18 (15.15)
**FIX**	10.00 (1.60) ^a^	28.34 (2.69)	37.71 (3.02)	45.99 (3.74)
**FIX EX**	38.39 (9.11)	94.23 (13.33)	117.52 (15.4)	134.37 (16.4)
**FII LC**	10.52 (1.69) ^a^	28.99 (2.16)	40.75 (2.63)	51.63 (2.70)
**DEX**	1.42 (0.41)	4.60 (0.56)	7.55 (0.69)	9.62 (0.75)
**BT**	0.76 (0.28)	2.77 (0.46)	4.43 (0.57)	5.95 (0.72)

For each material and considering each day (materials within the same vertical lines) mean values with the same minimal superscript letters don’t differ from each other at P = 0.05.

## Discussion

The content of fluoride in restorative materials should, however, be as high as possible without adverse effects on physical and mechanical properties and the release also should be as great as possible without undue degradation of the filling. An initial fluoride “burst” effect is desirable, as it will reduce the viability of bacteria that may have been left in the inner carious dentin and induce enamel/dentin remineralization.^20^ The reason for the rapid fall of fluoride release during subsequent days is likely to result from the initial burst of fluoride released from the glass particles as they dissolve in polyalkenoate acid during the setting reaction. Also, the high level of fluoride release on the first day may be caused by the initial superficial rinsing effect, while the constant fluoride release during the following days occurs because of fluoride ability to diffuse through cement pores and fractures.^21^ GC Fuji VII is a glass ionomeric filling material with additionally enriched ionic potential, namely strontium and calcium, a specially designed glass ionomer to control the active carious lesions in high risk patients.^22^ Fuji VII released the highest rate of fluoride compared to another tested materials in this study that can be in the same purpose of designing this material. Although a study results indicate that higher fluoride release of GICs was not able to reduce the amount of bacterial growth and biofilm formation on the surfaces of these materials when stored in natural saliva, based on another study results a monthly fluoride release consisting of 200-300 µg/cm^2^ is sufficient to completely inhibit enamel demineralization.^23^,^24^ The amount of 178.18 µg/cm^2^ measured released fluoride from Fuji VII in our study was related to 21 days release, and can gain the above-mentioned values (200-300 µg/cm^2^) during one month. The high level of F release on the first day may be caused by the initial superficial rinsing effect, while the constant F release during the following days occurs because of fluoride ability to diffuse through cement pores. In general, it may be supposed that there is a direct relationship between the fluoride present in the cement and the amount of fluoride released. The different chemical and physical characteristics of F VII and F IX may be responsible for their difference in fluoride release, as the results of this study about F VII and F IX, is in accordance with another study results.^21^ Low fluoride release in F IX is attributed to glass filler content with fewer monovalent ions cross linking the polymer chains holding them close together, leading to less water transport and, consequently less fluoride release.^25^ It is important to consider that different methodology used in the studies, including specimen size, media used to measure fluoride release and uptake, quantity of media used to measure fluoride and different methods to measure fluoride release are responsible for the high numerical differences found among studies.^22^,^26^–^28^ The highest values of cumulative fluoride release after Fuji VII was related to Fuji IX Extra, that can be related to incorporation of higher fluoride compounds compared to Fuji IX glass ionomer. Tay and colleagues^19^ found a thinner hydrogel layer in FII LC compared to thicker 300 nm silica gel layer in ChemFlex (Conventional glas ionomer) that became thicker upon water absorption and can be case for changing in trend of fluoride release in FII L after the first week of immersion. A very thin hydrogel layer in Dyract AP and no appreciable change occurred in Reactmer Paste by water storage. Higher amounts of fluoride release of FII LC compared to F IX on the first day of immersion and changing in trend of Fuji II LC for fluoride release is in accordance with the results of fluoride release by resin modified and conventional glass ionomers in another study^23^ and can be primarily due to ion exchange, but a degree of “wash-out” or dissolution may also contribute to the higher fluoride release. Although the amounts of fluoride released on the 1^st^ day by FII LC was greater than that of FIX, but their difference was not significant and is in accordance with the results of another study.^7^ Initial setting of resin modified glass ionomers is performed by light activated polymerization and is followed by an acid base reaction that arises from sorption of water. Resin modified glass ionomers were mostly found to have a potential for fluoride release in equivalent amounts as conventional cements, but may be affected not only by the formation of complex fluoride compounds and their interactions, but also by the type and amount of resin used for the photochemical polymerization reaction.^7^,^29^ Beautifil showed little amounts of controlled fluoride release in this study. Beautifil contains surface prereacted glass ionomer (S-PRG) as a fluoride component. The fluoride glass within Beautifil has little or no glass ionomer matrix phase, because of the lack of any significant acid base reaction. Since PGR has been prereacted with fluoroaluminosilicate glass and acid, water sorption is not critical in the acid base reaction as is seen in this study and is in agreement with the results of other studies.^19^,^30^ Another explanation for highly difference in fluoride release between GIC and resin composite like (compomers and giomers) is that, obviously the porosity of the materials may have a great influence on the amounts of fluoride release. Also, these materials have added resin contents compared to GICs, the barrier through which water and fluoride to diffuse also increases, in addition to their filler solubility differences.^28^ The porosity of the BT and DE is lower than that of tested GIC, so the fluoride release was not expected to be as much as the GIC. Dyract Extra also showed a low diffusion controlled fluoride release. Although Dyract includes a fluoride containing acid degradable glass and an acidic species capable of reacting with glass, there is no water present in the material to facilitate acid base reaction. If the reaction does occur, it is due to the diffusion of controlled uptake of water by the cement from the surroundings. In compomers, the functional groups of polyacid and methacrylates are combined into one molecule. Light curing results in a setting process analogous to that of composite resins. Subsequent water sorption leads to ionization of the acid groups and an acid base reaction resulting in fluoride release in a similar manner to that of the glass ionomers.^19^ The results of this study, partly is in accordance with another study results.^25^ Dyract Extra based on manufacturer information contains strontium fluoride, but it seems that incorporating this composition does not lead to much fluoride release from Dyract Extra compared to Dyract.^14^ With regard to compomers, several authors found differences in fluoride release in products with different filler systems. Compomers containing glass fillers and ytterbium trifluoride are reported to release higher amounts of fluoride than srF2 containing products.^7^ However, the difference between glass ionomers and compomers during the first week of immersion could be due to the fact that after curing and before contact with water, the fluoride in polyacid modified composite is not free, but bound in the filler particles, which are enclosed in the polymerized matrix and in the first phase of setting, polyacid modified composite resin completely behave like composites. Asmussen and Petzfeld^31^ found that compomers might release relatively little fluoride during the first year after setting, but thereafter, the rate of fluoride release become equal to that of glass ionomers.

Finally, a slow release of fluoride from dental materials may have clinical implications in vivo. Fluoride release from GICs restorations following a continuous uptake process increases the fluoride concentration in saliva and in adjacent hard dental tissues. Thus, continuous small amounts of fluoride surrounding the teeth decreases demineralization of the tooth tissues.^12^ Cate et al^32^ deduced that dentin demineralization was inhibited in a clinically relevant percentage only at fluoride levels above 1 ppm. Near optimum fluoride effects can be achieved with quite low concentrations in a daily fluoride rinse.^33^ The effect of a very low amount of continuous fluoride from giomers and compomers on dental hard tissues is needed to be further studied. Restorative materials with a high fluoride release generally have lower mechanical properties.^34^ Therefore, they may not be as durable clinically as lower fluoride releasing materials, particularly in load bearing areas. Mechanically stronger materials, usually release only a small amount of fluoride. Therefore, frequent external application of fluoride is necessary to maintain the high fluoride release and provide caries protection.

## Conclusion

Materials used in the present study all released fluoride, but a higher rate in fluoride release was seen for Fuji IX, Fuji VII, Fuji IX Extra, Fuji II LC compared to BT and DEX. DEX and BT both released low amounts of fluoride, but DE released more fluoride compared to BT. It seems that the extent of the glass ionomer matrix plays an important role in determining the fluoride releasing ability of GICs materials.
